# My Sixth Case of Placenta Prævia

**Published:** 1892-06

**Authors:** Edmund Carleton


### MY SIXTH CASE OF PLACENTA PRÆVIA.

Prof. Edmund Carleton, M. D.

Read before the International Hahnemannian Association, Richfield Springs, June, 1891.

The subject of this sketch was the mother of a number of children. All former pregnancies had been natural and easy. No hemorrhage occurred before term in this instance. I was summoned in the evening of April 17th, 1891. First discovered blood and clots; then the os dilated about one inch; the placenta covered it, but the examining finger found that the thinnest part extended anteriorly. During pains I carefully pushed my finger up to the thin edge and diagnosed a head presentation. Blood did not come so rapidly, but that it mostly clotted. I dissolved Pulsatilla$^{200}$ in water, and gave a teaspoonful every ten minutes. Pains came on rapidly and forcibly. As dilatation advanced, I pushed the placenta backward with my finger, which allowed the head gradually to come down and finally to engage occiput right anteriorly. Uterine contractions and my pressure did the tamponing, so that hemorrhage never became alarming; and as soon as the head engaged, bleeding nearly ceased.

When the amniotic fluid escaped, it was with a rush which brought down an arm beside the head, and in that relation it was born, the child being small. The placenta was delivered soon after. Contractions firm. The whole affair was over in about an hour and a half. Patient not exsanguinated, and got up quickly.

The child (male) appeared to be dead. Persistent artificial respiration, kept up for thirty minutes, with friction and heat, saved his life.

This is my sixth case of placenta prævia, and I hope the last. Five mothers and four children survived. Much has been said and written on the subject and different theories advocated. As the line of conduct indicated in this paper is the one that I have always followed, please to consider it as the exemplification of my views.

